# The role of FOXA family transcription factors in glucolipid metabolism and NAFLD

**DOI:** 10.3389/fendo.2023.1081500

**Published:** 2023-01-31

**Authors:** Chuchu Yu, Xiaojing Li, Yu Zhao, Yiyang Hu

**Affiliations:** ^1^ Key Laboratory of Liver and Kidney Diseases (Ministry of Education), Shanghai Key Laboratory of Traditional Chinese Clinical Medicine, Institute of Liver Diseases, Shuguang Hospital Affifiliated to Shanghai University of Traditional Chinese Medicine, Shanghai, China; ^2^ Institute of Clinical Pharmacology, Shuguang Hospital Affifiliated to Shanghai University of Traditional Chinese Medicine, Shanghai, China

**Keywords:** forkhead box, glucose metabolism, lipid metabolism, age-related metabolism, NAFLD

## Abstract

Abnormal glucose metabolism and lipid metabolism are common pathological processes in many metabolic diseases, such as nonalcoholic fatty liver disease (NAFLD). Many studies have shown that the forkhead box (FOX) protein subfamily FOXA has a role in regulating glucolipid metabolism and is closely related to hepatic steatosis and NAFLD. FOXA exhibits a wide range of functions ranging from the initiation steps of metabolism such as the development of the corresponding metabolic organs and the differentiation of cells, to multiple pathways of glucolipid metabolism, to end-of-life problems of metabolism such as age-related obesity. The purpose of this article is to review and discuss the currently known targets and signal transduction pathways of FOXA in glucolipid metabolism. To provide more experimental evidence and basis for further research and clinical application of FOXA in the regulation of glucolipid metabolism and the prevention and treatment of NAFLD.

## 1 Introduction

Hepatic lipid metabolism and glucose metabolism are closely related and they go hand in hand in the pathogenesis of NAFLD (1). Currently, 25% of the world’s population is considered to have NAFLD, and NAFLD has evolved to become the leading cause of chronic liver disease worldwide (2). NAFLD is caused by the expansion of fat depots and accumulation of ectopic fat causing insulin resistance. In this condition, inappropriate lipolysis leads to an unabated transport of fatty acids to the liver, accompanied by an increase in *de-novo* lipogenesis, leading to cellular stress such as oxidative stress and endoplasmic reticulum stress (3, 4). The pathological process of NAFLD also includes other hepatic lipid metabolic pathways such as triglyceride (5) and bile acid metabolism (6). In addition, NAFLD is closely related to other metabolic diseases such as type 2 diabetes. They share the same key pathogenic factors such as obesity and insulin resistance and therefore often coexist. There are synergistic effects between these two diseases that increase the risk of adverse clinical outcomes (7). Therefore, it is urgent to correct abnormal glucolipid metabolism.

**Figure 1:**
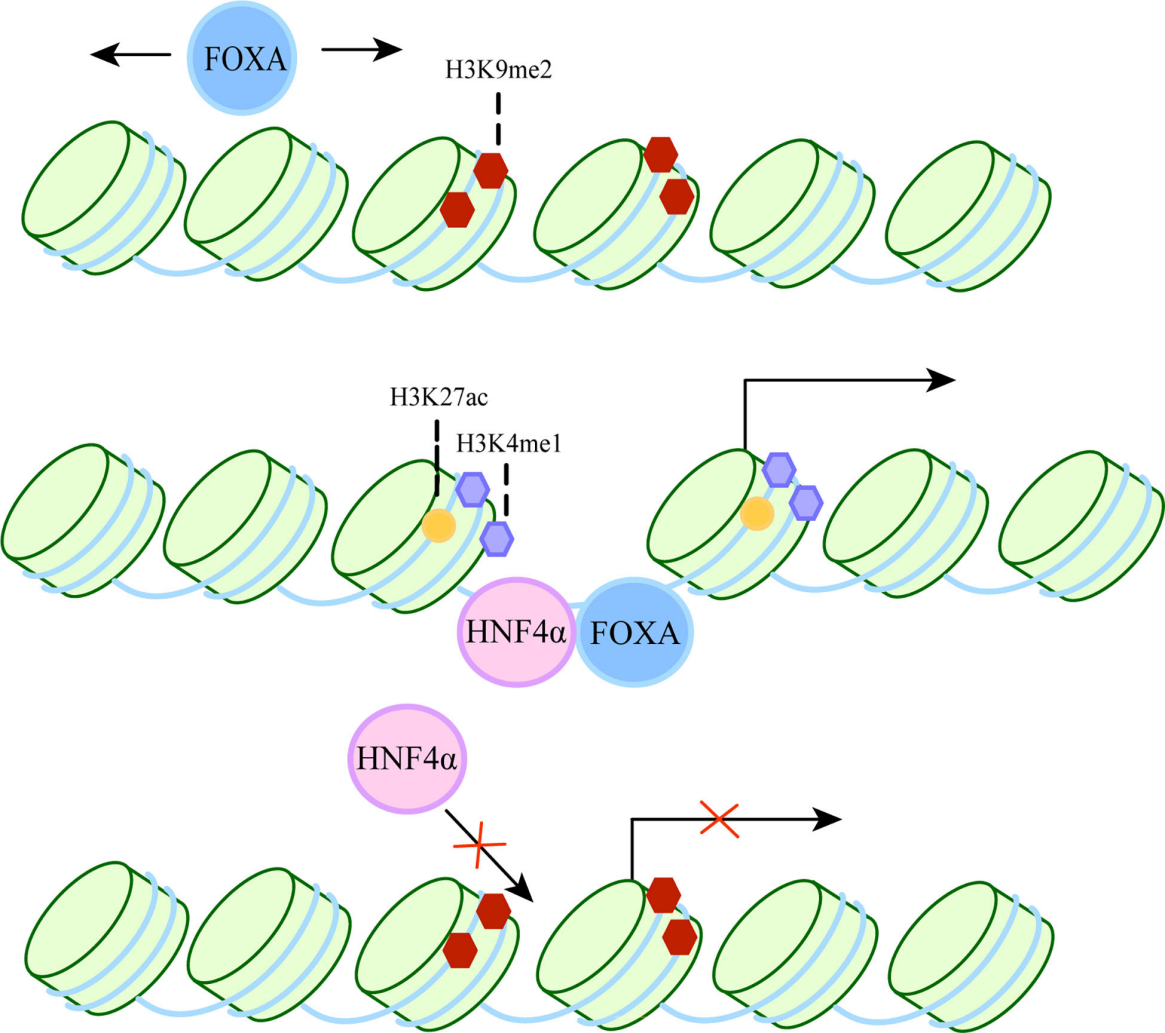
The dual role of FOXA as a pioneer and settler transcription factor. FOXA can bind target DNA sequences around nucleosomes in condensed chromatin. The first step, FOXA scans the chromatin laterally (12) and uses short anchoring α-helix structures to identify DNA motifs (13). The second step, since the crystal structure of the DBD of FOXA is highly similar to the ligand histone, they both use a “winged helix” structure to bind to DNA along the long axis of DNA (10, 14). In some cases, FoxA can replace linker histones (15). Then, induction of nucleosome repositioning and recruitment of co-transcription factors such as hepatocyte nuclear factor 4α (HNF4α), followed by high chromatin accessibility and aggregation of methylated H3K4 (H3K4me1) and acetylated H3K27 (H3K27ac). The resulting permissive chromatin state promotes transcriptional initiation, as FOXA cooperates with HNF4α to activate liver-specific genes (16).

FOXA belongs to the Forkhead box protein family, which includes FOXA1, FOXA2 and FOXA3 (8, 9). It has been shown to act as a “pioneer factor” (10) by binding to inactive regions of chromatin and inducing changes in chromatin accessibility, thereby facilitating the binding of other transcription factors. It also acts as a “settler” transcription factor, maintaining an open isochromatin state after unlocking chromatin to prevent chromatin closure and hindering the binding of transcriptional regulators (11). The specific process is shown in **Figure 1**. Therefore, they can participate in a variety of complex cellular processes and play an important role in many diseases, such as liver fibrosis (17), hepatocellular carcinoma (18).

There is now growing evidence that the FOXA family plays a role in the development of organs closely related to metabolism: the liver and pancreas, and that the FOXA family also has a complex and extensive network of regulatory roles in multiple aspects of glucose and lipid metabolism, including glucagon and insulin secretion, fatty acid and triglyceride metabolism, and several pathogenic processes of NAFLD as described above. FOXA proteins have also been associated with age-related aspects of metabolic regulation. The aim of this paper is to review and highlight the functions of FOXA transcription factors in glycolipid metabolism and metabolic diseases such as NAFLD. In addition, we focus on microRNAs (MiRNAs) that can target FOXA and other approaches that have an impact on its activity and function and may help prevent or treat glucolipid metabolic diseases such as NAFLD.

## 2 Naming, structure and expression of FOXA

**Figure 2:**
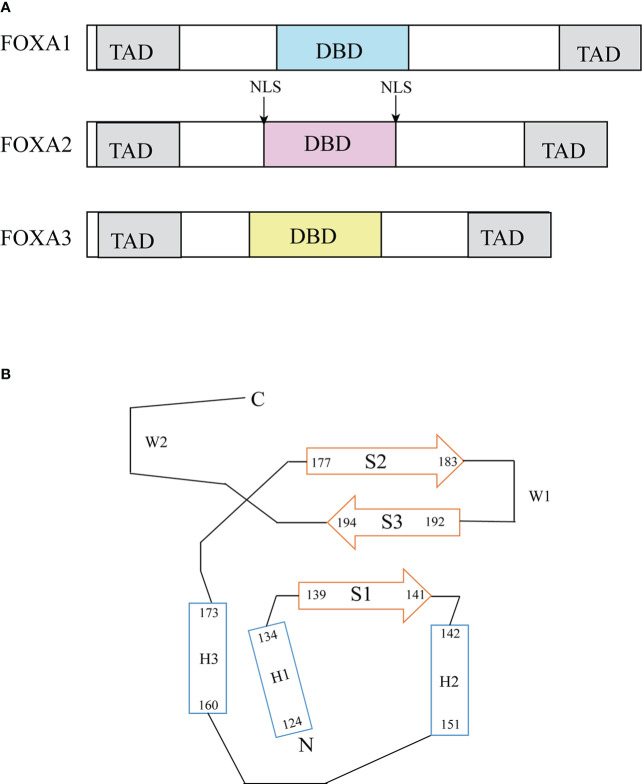
Structure of FOXA. **(A)** Functional domains of FOXA family (23). FOXA1, 2 and 3 have substantial homology in the DNA binding domain, FOXA1 has 95% sequence homology with FOXA3 and 90% homology with FOXA2 (24). **(B)** Topology of FOXA3 DNA binding domain (24). DBD, DNA-binding domain; TAD, transactivation domain; NLS, nuclear localization signals.

The FOXA protein family was originally discovered based on the presence of DNA binding activity in hepatocyte nuclear extracts (19). For this reason, these genes were initially named hepatocyte nuclear factor-3 (HNF-3) α, Β and γ until 2000 when the nomenclature was standardized to FOXA1, 2, 3 (20). These proteins are named forkhead box because they contain a winged helix structure necessary for binding to the target DNA, the structure consists of three alpha-helices arranged in a helix-turn-helix core flanked by rings (21). Two polypeptide chains flanking the DNA binding domain responsible for nuclear localization; and conserved transactivated structural domains at both ends (22) (**Figure 2**). The FOXA protein family express in multiple organs and tissues in humans and mice, such as the liver, gastrointestinal tract, etc (23). (**Table 1**)

**Table 1:** The FOXA protein family express in multiple organs and tissues in humans and mice.

	Adult mouse			Human embryo			Adult human		
	FOXA1	FOXA2	FOXA3	FOXA1	FOXA2	FOXA3	FOXA1	FOXA2	FOXA3
Brain									√
Endocrine tissues									√
Bone marrow & immune system							√	√	√
Muscle tissue									√
Lung	√	√	√	√	√		√	√	√
Liver and gall bladder	√	√	√	√	√	√	√	√	√
Pancreas	√	√			√			√	√
Gastrointestinal tract	√	√	√	√	√	√	√	√	√
Kidney & urinary bladder	√						√		√
Male reproductive tissues	√		√				√	√	√
Female reproductive tissues							√	√	√
Adipose & soft tissue									√
Skin									√

## 3 Role of FOXA on the development of liver and pancreas

The liver and pancreas are important organs involved in metabolic processes such as glucose and lipid metabolism. FOXA1 and FOXA2 act together in foregut endoderm and are required for the normal development of endodermal origin organs such as the liver and pancreas (21).

### 3.1 The development of liver

FOXA1 gene-inactivated mice died early in life, but their livers appeared histologically normal (25), the livers of FOXA3 mutant mice were also apparently normal (26). Mice with specific deletion of endodermal FOXA2 also exhibited normal growth of the liver. Thereby demonstrating that a single deletion of FOXA1, FOXA2 or FOXA3 does not prevent the establishment of hepatic developmental capacity. However when FOXA1 and FOXA2 were simultaneously absent from the foregut endoderm, liver identity was completely blocked and these embryos completely lacked liver specification, with neither liver bud development nor expression of the earliest hepatic marker gene, alpha-fetoprotein (AFP) and hepatoblast markers albumin and transthyretin (27, 28). In addition, FOXA1 and FOXA2 co-induce albumin expression with Gata4, providing a preliminary specification for liver differentiation (29). Similarly, the role of FOXA2 in human liver endoderm differentiation has been demonstrated. The loss of FOXA2 resulted in a significant reduction in the expression of hepatic endodermal transcription products such as ALB and important transcription factors involved in liver development including HNF4a and HHEX (30).

After liver specification, hepatocyte differentiation was greatly independent of FOXA1/2 (31). However, Yitzhak Reizel et al. depleted three FOXA genes in the livers of adult mice exhibiting liver failure and liver inflammation, demonstrating that the critical role of FOXA protein is not limited to early organ development. FOXA also acts as “settlers” in the adult liver by promoting the binding of HNF4α to enhancers, thereby stabilizing the expression of some developmentally induced genes and the adult liver regulatory network (32). This is consistent with the previous report of significant overlap between HNF4a and FOXA2 binding regions in hepatocytes (33). Once the FoxA doorstop is removed, it results in the loss of chromatin accessibility at FoxA-binding sites and prevents continued binding of HNF4α, as evidenced by loss of hepatic identity (11) (**Figure 1**).

### 3.2 The development of pancreas

FOXA1, 2, and 3 are produced in the foregut endoderm at the beginning of pancreatic development, and continue to be expressed into adulthood (34). During pancreatic genealogy specification, FOXA2 is required to establish the initiation enhancer state and the correct remodeling of chromatin structure to recruit additional transcription factors, such as GATA6, for the activation of the transcriptional program required for human pancreatic differentiation. FOXA2 particularly affects the transition from the hind foregut (FG) to the primary pancreatic progenitor (PP1) stage (35). Activation of Pdx1 is a marker of normal pancreatic development in the foregut endoderm, FOXA1 and FOXA2 jointly occupy multiple regulatory domains of the Pdx1 gene, and compound mutation of FOXA1 and FOXA2 in the pancreatic primordia leads to complete loss of Pdx1 expression, failure of pancreatic primordia expansion, blocked exocrine and endocrine cell differentiation, and other signs of severe pancreatic dysplasia (36).

## 4 Regulation of glucose metabolism

### 4.1 Β cell

Mice with islet-specific deficiency of FOXA1 exhibited decreased glucose-stimulated insulin release and reduced pancreatic insulin content. Mutant islets required higher glucose levels to initiate the first-phase response. The intrinsic reason for this phenomenon is that deficiency of FOXA1 causes increased expression of the uncoupling protein 2 (UCP2), which results in mitochondrial oxidative phosphorylation being partly uncoupled, the change in glucose concentrations is no longer efficiently converted to increased ATP levels, further affecting the subsequent closure of ATP-dependent potassium channels and the inward flow of calcium ions, ultimately inhibiting insulin release. In conclusion, although FOXA1 is not required for the initial specification of pancreatic Β cell, the terminal differentiation to fully mature Β cell is dependent on FOXA1 expression (37).In addition, lack of Groucho-related gene3/4-mediated repression leads to ectopic expression of FOXA1, which represses the expression of the Β cell transcription factor gene Neurod1, leading to loss of Β cell proliferation, early lethality, and hyperglycemia (38).

Consistent with genetic screening results in patients with congenital hyperinsulinemia (39), FOXA2 inactivation in mature Β cells induced hyperinsulinemia and hypoglycemia. After glucose withdrawal, the mutant islets took longer to reduce insulin secretion to baseline. The main mechanism is that impaired intracellular calcium oscillations and elevated cAMP levels may contribute to increased membrane-docked pools of readily released insulin granules and subsequent exocytosis (40). Similarly, hyperinsulinemic hypoglycemia was seen in 8-day-old mice with FOXA2-specific deletion of Β cells and resulted in premature death after birth. In addition, the mRNA levels of the genes encoding Kir6.2 and SUR1, the two subunits of the K-ATP channel, were significantly reduced in this model, and the defective expression of the K-ATP channel may be a cause of abnormal insulin secretion and hypoglycemia in these mice (41). Since miR141 was upregulated in both diabetic mice and diabetic elderly patients, XIN YU et al. found that overexpression of miR141 leads to impaired glucose-stimulated insulin secretion and damaged Β cell proliferation. In addition to pioglitazone to correct this process, FOXA2, the direct target of miR141, may also play a role in reversing it (42). To better understand the role of FOXA2 in Β cell development, Ahmed K. Elsayed et al. generated induced pluripotent stem cells (iPSCs) from a patient with FOXA2 haploinsufficiency (FOXA2^+/-^) and subsequently underwent Β cell differentiation. They found that FOXA2 haploinsufficiency downregulates gene expression essential for Β cell differentiation and normal function and upregulates genes related to neuronal development, the WNT and BMP pathways, negatively affecting endocrine islet development (43).

**Figure 3:**
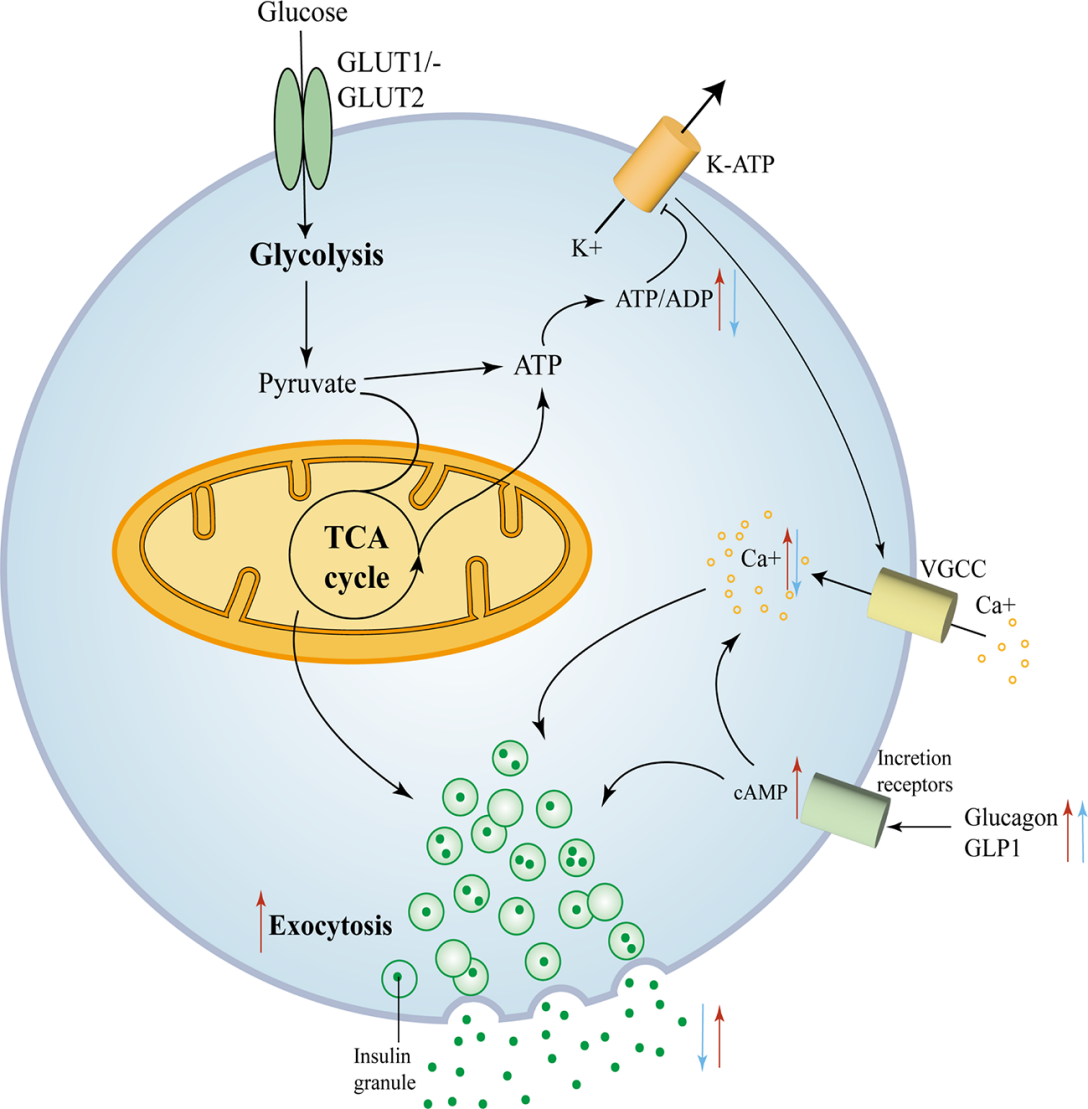
FOXA1 and FOXA2 control insulin secretion in mature Β cells. FOXA1 and FOXA2 control insulin secretion in mature Β cells. Glucose enters the Β cell and moves into the glycolytic pathway and is then oxidized through the tricarboxylic acid (TCA) cycle and subsequently produces ATP, with an elevated ATP/ADP ratio leading to the closure of KATP channels, membrane depolarization and subsequent opening of voltage-gated calcium channels. The consequent trigger phase of insulin granule cytokinesis is driven by an increase in intracellular calcium produced due to the opening of calcium channels (45). The red arrows mark key components of glucose metabolism and insulin secretion that are affected by the removal of FOXA2. The blue arrows mark key components that are affected by the removal of FOXA1.

Compound mutations had more pronounced hypoglycemia and hyperinsulinemia than FOXA2 mutations alone, suggesting that FOXA2 appears to be a more potent regulator than FOXA1 (44). The mechanisms of the effect of FOXA1 and FOXA2 deficiency on Β cell insulin secretion are depicted in **Figure 3**.

### 4.2 α cell

FOXA1 and FOXA2 control glucagon biosynthesis and secretion and α cell differentiation through common and unique target genes. Consistent with the reduction in glucagon gene expression levels, the content of glucagon was reduced in FOXA1-, FOXA2-, and FOXA1/FOXA2-deficient α cells, and it seems that only the combined silencing of FOXA1 and FOXA2 can affect the secretion of glucagon (46).

In endoderm-specific FOXA2 knockout mice (FOXA2loxP/loxP; FOXA3Cre) (47), the initial steps of endocrine cell differentiation occurred normally, however, α cells did not complete terminal differentiation, as evidenced by a dramatic decrease in the number of mature α cells, leading to hypoglucagonemia and hypoglycemia as well as early mutant death.

In addition to the above-mentioned specific mutant of FOXA1 and FOXA2 in mouse pancreatic islets, mice lacking FOXA1 generated by homologous recombination in embryonic stem cells also exhibited persistent hypoglycemia and early postnatal death, etc. On the premise that pancreatic α and Β cells from mutant mice have been shown to differentiate normally, hypoglycemia in mice did not stimulate glucagon secretion, and its level paradoxically decreased. This phenomenon suggests that α cells of FOXA1-deficient mice lose the ability to respond to hypoglycemia by increasing glucagon secretion. The mutant mice showed significantly higher blood glucose levels and lower serum insulin concentrations after receiving intraperitoneal glucose stimulation, suggesting that FOXA1 is also essential for normal islet Β cell function (48).

### 4.3 Regulation of the G1,G2 element of the glucagon gene promoter

All three FOXA proteins bind to the G2 element of the glucagon gene promoter (25, 46) (49–51). FOXA1 is the most potent transactivator *via* the G2 element, but the role of FOXA2 binding to the G2 element on glucagon seems to be controversial, FOXA2 and G2 element binding is important for glucagon gene promoter activity and gene transcription, but JACQUES PHILIPPE et al. (51) proposed that FOXA2 and G2 element binding acts as a repressor of glucagon gene expression. In addition, FOXA3 is not essential for controlling islet or intestinal glucagon gene expression.

**Figure 4:**
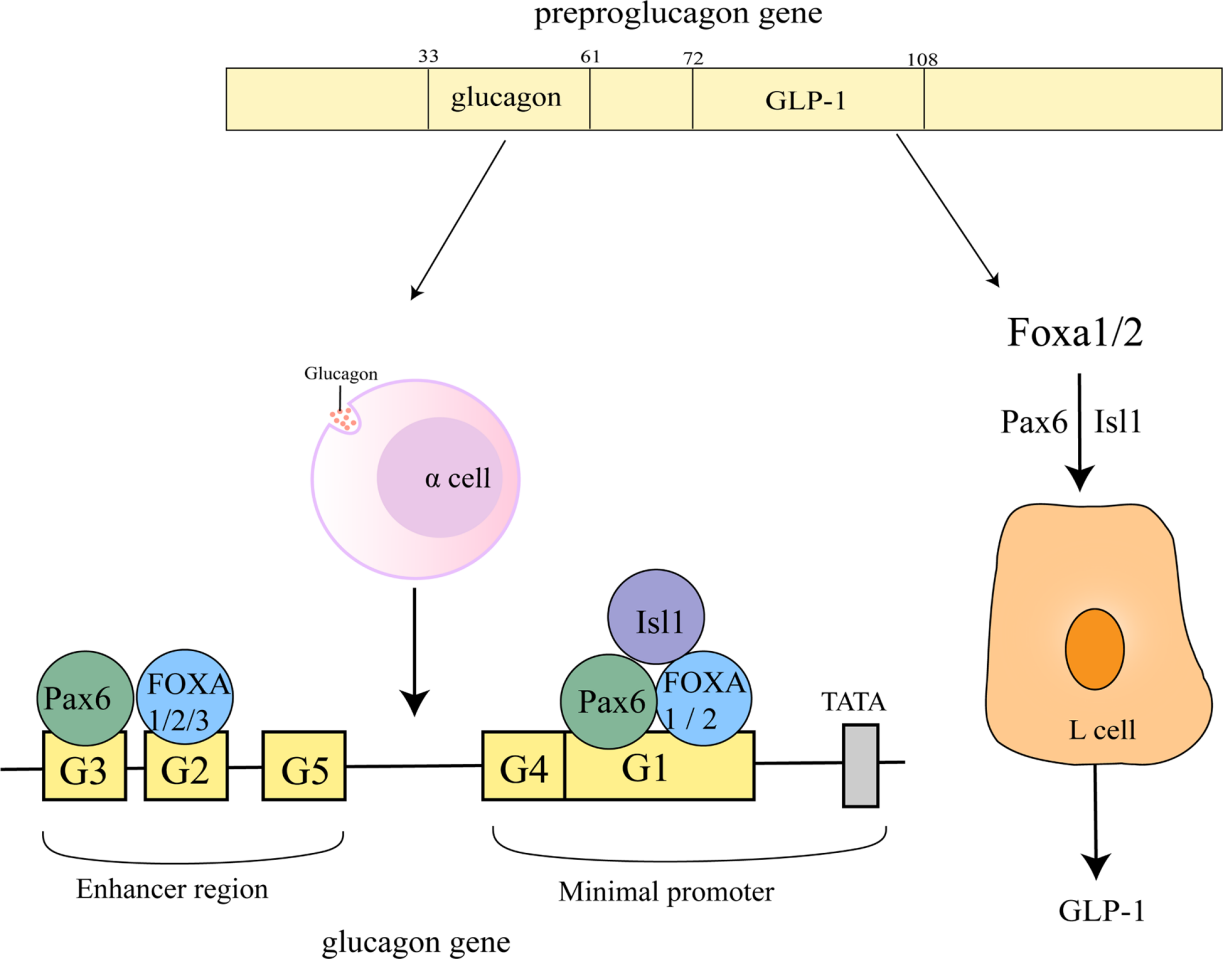
Regulation of glucagon gene promoter and GLP-1 by FOXA. The sequences of glucagon and GLP-1 correspond to 33-61 and 72-108 of the proglucagon sequence (53). On the glucagon gene promoter, FOXA1 and FOXA2 bind to the G1, G2 element, FOXA3 is located on the G2 element, Pax6 is located on the G1, G3 element and Isl1 on the G1 element. FOXA1 and FOXA2 act upstream of ISL-1 and Pax6 to regulate L-cell differentiation, control GLP1 production.

FOXA1 and FOXA2 may play a dual role in glucagon gene transcription by inhibiting the activation of Pax6 on G1 and G3 elements to attenuate its transactivation potential at the glucagon gene promoter and through direct activation by G1. Of these two, FOXA2 appears to be the primary binding activity detected on the G1 element. Not a single one of G1 and G2 can be omitted in the process that maximal activation of the glucagon gene promoter by FOXA1/2 (52) (**Figure 4**).

### 4.4 Regulation of GLP-1

Glucagon-like peptide-1 (GLP-1) can control meal-related blood glucose fluctuations by enhancing insulin and inhibiting glucagon secretion (54). FOXA1 and FOXA2 act upstream of ISL-1 and Pax6 to regulate L-cell differentiation, control GLP1 production and disrupt preproglucagon transcription (**Figure 4**) (55). miR-194 inhibits GLP-1 synthesis in L cells by suppressing FOXA1-mediated transcription of pcsk1. In contrast, miR-194 silencing in HFD-induced obese mice significantly increased the levels of FOXA1, Pcsk1 and GLP-1, and both oral glucose tolerance and insulin tolerance were also attenuated (56).

### 4.5 Hepatic glucose metabolism

The liver regulates glucose uptake and storage through gluconeogenesis and glycolysis, and glucose production and release through glycogenolysis and gluconeogenesis, thereby controlling the balance of glucose levels between blood flow and hepatocytes (59).

FOXA1/A2 and FoxO1, which is the other transcription factor of the FOX family, bind interdependently at the insulin response element (IRE) and synergistically regulate the expression of two insulin-regulated genes, IGFBP1 and G6PC (60). FOXO1 and FOXA2 form a complex on chromatin at the IGFBP1 promoter in HepG2 cells and cooperate to open linker histone H1 compacted IGFBP1 promoter nucleosome arrays (61). In addition, under fasting or diabetic conditions, glucagon upregulates TET3 above a critical threshold level, so that TET3 can be recruited to the P2 promoter of HNF4α by FOXA2. The binding induces P2 promoter demethylation, which further activates PICK, G6PC and contributes to gluconeogenic activation (62). Glucagon can also inhibit the ability of FXR to induce the anti-gluconeogenic nuclear receptor SHP by activating FOXA2 (63). LiPing Zhang et al. (64) also demonstrated that FOXA2 is required to execute the hepatic gluconeogenesis procedure by integrating the transcriptional response of hepatocytes to hormonal (glucagon, glucocorticoid) stimulation during fasting. In order to increase FOXA2 expression in mouse liver, both mice injected with adenovirus in the tail vein (65) and prepared T-77 transgenic mice (66) exhibited a decrease in liver glycogen stores and a transient decrease in serum glucose levels. Transgenic mice maintained adequate serum glucose levels for survival by utilizing transiently elevated lipid levels, and also undergoing gluconeogenesis using deaminated amino acids with dicarboxylate products of peroxisomal lipid Β-oxidation shuttled through the tricarboxylic acid cycle.

In terms of glycolysis, it was shown that FOXA2 binds directly to the gene promoter of glycolytic enzymes (67). Moreover, in hepatic progenitor cells, FOXA2 inhibits PI3K/Akt-regulated HK2 activity to suppress aerobic glycolysis and further inhibits cell proliferation (68).

Vitro studies (69) have shown that FOXA3 activates the transcription of important gluconeogenic genes: glucose-6-phosphatase (G6pase). However, FOXA3-deficient mice showed no reduction in hepatic PEPCK or G6pase expression after 24 to 72 hours of fasting when blood glucose levels decreased. In contrast, hepatic glucose transporter protein 2 (Glut2), the promoter of which has been shown to contain a FOXA binding site (70), was significantly reduced in FOXA3^-/-^ mice, which may affect hepatocyte glucose output leading to a hypoglycemic phenotype during prolonged fasting (71).

## 5 Regulation of lipid metabolism

A series of experiments has shown that the expression of FOXA is altered in a variety of animal models and patients with steatosis. FOXA1 mRNA levels were lower in the livers of NAFLD patient groups and MCD diet-fed steatosis rats, and FOXA1 significantly reduced steatosis in human hepatocytes and HepG2 cells cultured with oleic and palmitic acids (72); FOXA2 expression was significantly reduced in liver tissue of patients with steatosis or NASH (73), but the expression of FOXA2 was increased in both HFD-fed mice and fa/fa rat models, and its levels were corrected by the insulin-sensitizing medication pioglitazone (74). Genetic data suggests a potential relationship between the FOXA3 gene and the human metabolic phenotype (75). Consistent with this, FOXA3 expression was elevated in the liver of both NAFLD patients and HFD-fed mice (76). The existing mechanisms of how the FOXA family regulates lipid metabolism and causes steatosis are as follows.

### 5.1 Adipocyte differentiation

FOXA1 gene expressed in preadipocytes (77), its expression was highest at 3h after the onset of adipocyte differentiation and then diminished gradually, and FOXA1 inhibited lipid accumulation and the expression of adipose genes such as PPARγ under the regulation of C/EBPΒ (78). FOXA2 was undetectable in adipose tissue of lean mice, but expressed in adipose tissue of diet-induced obese mice or genetically obese mice. FOXA2 blocks adipogenesis in preadipocytes by inducing Pref1, thereby inhibiting the induction of late markers of adipocyte differentiation such as PPARγ, aP2 (79).

According to the study of two models of adipocyte differentiation *in vitro*, Lingyan Xu et al. proved that different from FOXA1\FOXA2, FOXA3 is a positive regulator of adipocyte differentiation. FOXA3 promotes adipocyte differentiation by inducing PPARγ expression. This process is accomplished by the synergistic transcription of FOXA3 with C/EBPΒ and C/EBPδ. HFD selectively induced FOXA3 in epithelial fat, while removal of FOXA3 in mice prevented weight gain and the development of visceral obesity, as well as adipocytes obtained from visceral fat expressing lower levels of PPARγ and differentiation markers (80). FOXA3 can increase fat accumulation by increasing the activation of the lipid storage mechanism in selective fat depots only when food is abundant, suggesting that FOXA3 may also act as a “hoarder” gene (81).

### 5.2 Regulation of fatty acid (FA) metabolism

#### 5.2.1 Fatty acid uptake and transport

Marta Moya et al. (72) showed that both Ad-FOXA1-transfected HepG2 cells and human hepatocytes exhibited decreased FA uptake and increased percentage of residual FA in the culture medium. Similarly, the major contributor to FA uptake, FA transporter protein (FATP2), was decreased in both types of cells compared to controls.

Dietary triglycerides (TG) undergo a series of emulsification and hydrolysis in the intestinal lumen before being taken up by enterocytes and resynthesized into triglycerides. Chylomicrons transport these TG to the plasma. Due to lipoprotein lipase activity, most of the chylomicron TG is taken up by muscle and adipose tissue, and triglycerides remaining in the chylomicron remnants are transported to the liver, becoming one of the sources of FA in hepatocytes (58). Inhibition of LPL activity promotes hepatic uptake of triglycerides from chylomicron remnants, leading to the development of hepatic steatosis (4). Maria Kanaki et al. demonstrated the presence of FOXA2 binding sites on the human LPL gene promoter and the dose-dependent activation of the LPL promoter by FOXA2. In addition, FOXA2 nuclear translocation caused by phosphorylation of AKT kinase during insulin treatment also affects LPL expression (82). Since LXR can induce LPL and there are physical and functional interactions between LXRα and FOXA2, silencing of FOXA2 by siRNAs or insulin treatment not only suppresses basal expression of LPL genes, but also abolishes LXR agonist-induced expression of LPL genes (83). The effect of FOXA2 on LPL is clear, but further studies are needed to determine whether it can influence fatty acid uptake and liver steatosis through this pathway.

#### 5.2.2 Fatty acid oxidation

The main pathway for most FA oxidation in hepatocytes is mitochondrial beta oxidation (58). FOXA2 phosphorylation by Insulin–Irs1/Irs2-PI3Kinase–Akt signaling leads to nuclear export (67, 84), and the nuclear export is positively correlated with insulin concentration. In three insulin-resistant mouse models: ob/ob mice, lipoatrophic aP2-nSrebp-1c (Srebp-1c) mice, and high-fat-induced obese mice, adenoviral expression of FOXA2T156A, the FOXA2 that cannot be inhibited by insulin, in addition to increased nuclear localization of FOXA2, decreased weight, hepatic triglyceride content, reduced glucose production and plasma insulin, increased hepatic insulin sensitivity, Β-oxidation and ketone body production. Consistent with these changes, FOXA2 affects genes of the corresponding metabolic processes and has been shown to bind directly to promoters of genes encoding Β-oxidation, ketogenesis (67).

Glucagon signaling and fasting activate adenylate cyclase (AC), which inhibits SIK2, a negative regulator of p300 activity. p300 can acetylate FOXA2, thereby blocking its nuclear rejection and increasing its transcriptional activity, which further led to increased mitochondrial oxidation, hepatic ketogenesis, insulin sensitivity and attenuated hepatic steatosis in db/db mice. Accordingly, phosphorylation and acetylation of FOXA2 appear to have opposite effects on the nuclear localization and transcriptional activity of FOXA2, and the predominance of acetylation over phosphorylation was demonstrated by double mutation of the acetylation site and phosphorylation site (85). Blocking the phosphorylation of FOXA2 or promoting its acetylation may be an attractive therapeutic target for the treatment of NAFLD and diabetes mellitus

In addition, the livers of FOXA2^-/+^ mice showed significantly reduced ketone synthesis and Β-oxidation, and this effect was more evident when the mice were given high-fat diet, directly demonstrating the effect of FOXA2 on ketogenesis and Β-oxidation (67). Although FOXA2 can activate mitochondrial Β-oxidation genes and enhance fatty acid metabolism alone, the effect of FOXA2 is further amplified by PPARγ activator Β (PGC-1Β), as PGC-1Β interacts with FOXA2 and enhances transcriptional activation of FOXA2 (86). Even in Huh7.5 cells, ectopic expression of FOXA2 rescued the decreased expression of medium-chain acyl coenzyme A dehydrogenase (MCAD) and short-chain acyl coenzyme A dehydrogenase (SCAD) involved in the regulation of fatty acid oxidation due to hepatitis C virus infection, and FOXA2 also reduced the formation of lipid droplet due to infection (87).

FOXA1 activates ketogenesis by inducing mitochondrial 3-hydroxymethylglutaryl coenzyme A synthase (HMGCS2), and both Ad-FOXA1-infected HepG2 cells and human hepatocytes displayed increased levels of ketone bodies (72). FOXA2 knockdown also led to a significant reduction in HMGCS2 expression, and miR-29 acted as an “intermediate bridge” that is a feedforward micro-regulator of HMGCS2. miR-29 levels in hepatocytes are regulated by FOXA2 but miR-29 can also modulate FOXA2-mediated regulation of key genes in lipid metabolism (74). Therefore, investigation of the integrated network of miRNAs and FOXA proteins may provide a better strategy for improving glycolipid metabolism.

**Figure 5:**
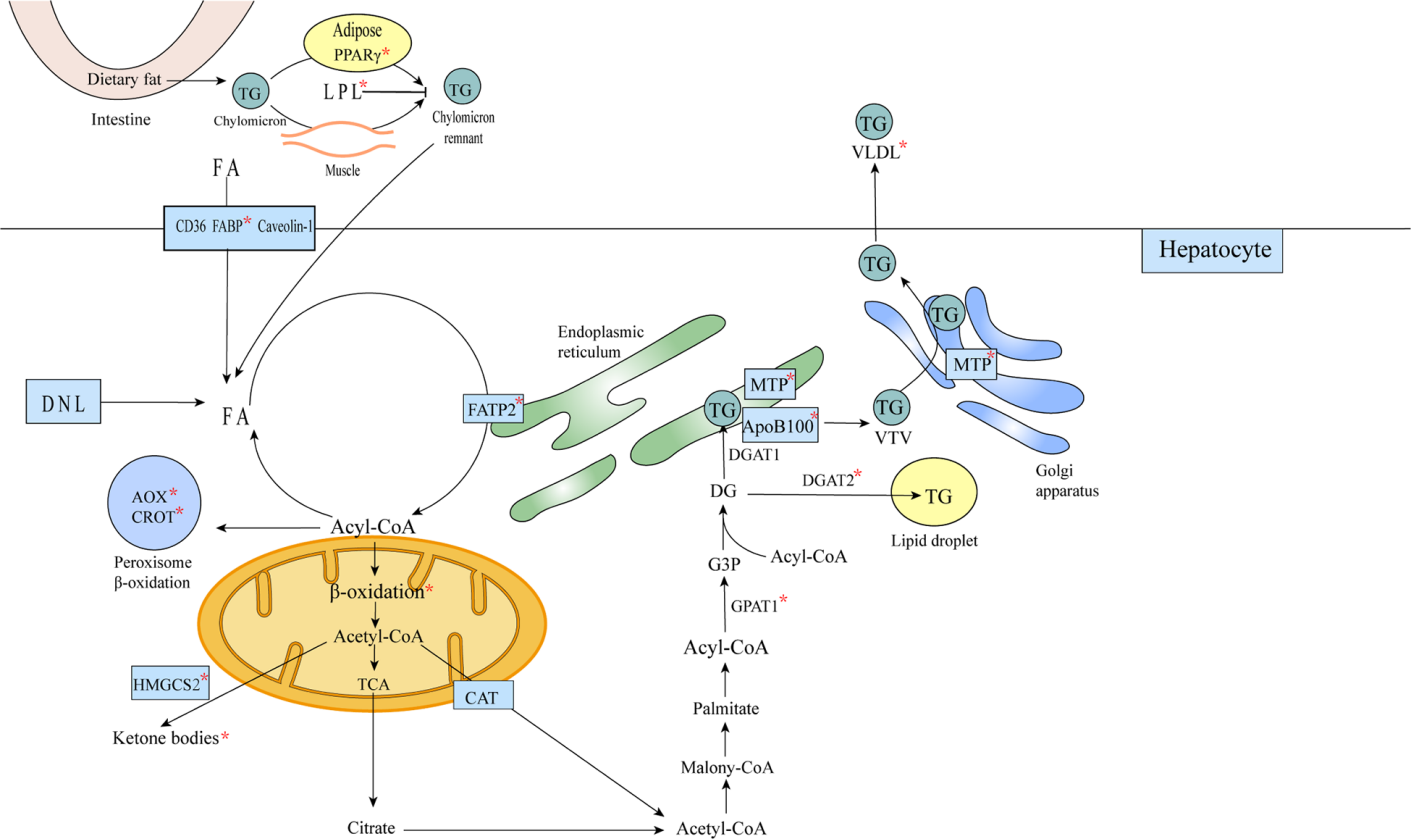
FOXA affects lipid metabolism in hepatocytes. Triglycerides remaining in the chylomicron remnants are transported to the liver as a source of FA in hepatocytes, and FA translocase (FAT)/CD36, plasma membrane FA-binding protein (FABP) and Caveolin-1 mediate the uptake of fatty acids bound to circulating albumin, or the acquisition of FA through *de novo* lipogenesis (DNL). Acetyl coenzyme A is converted to citric acid, ketone bodies or to carnitine/acetyl coenzyme A (CAT), from the mitochondria into the cytosol (57). In the cytosol, acetyl coenzyme A is used for the synthesis of triglycerides and very low density lipoproteins. Triglycerides can be stored in intracellular lipid droplets or packaged into very low density lipoproteins and secreted into the plasma. The formation of mature VLDL requires two lipidation processes mediated by MTP (58).The metabolic processes or substances involved in metabolism affected by FOXA are marked with an asterisk in the figure.

Β-oxidation of fatty acids also occurs in the peroxisome, except in the mitochondria (88). FOXA1 induced increased expression of rate-limiting enzymes in the peroxisomal Β-oxidation pathway and significant upregulation (14-fold) of genes involved in peroxisomal Β-oxidation (72) (**Figure 5**).

### 5.3 Regulation of triglyceride metabolism

Some genes involved in the TG synthesis pathway (GPAT1, DGAT2) were significantly inhibited by FOXA1 in human hepatocytes and HepG2 cells. The TG metabolism is closely related to the VLDL secretion, and most genes involved in VLDL synthesis (MTP, ACAT2 APOB) were also significantly downregulated. Furthermore, when Ad-FOXA1 infected human hepatocytes and HepG2, both intracellular and secreted ApoB100 proteins were significantly reduced. Thus, it is suggested that FOXA1 and TG synthesis and secretion are related (72).

The effect of FOXA2 in reducing hepatic TG content and increasing plasma TG concentration was increased by PGC-1Β, and FOXA2/PGC-1Β induced microsomal transporter protein (MTP) expression, thereby increasing the secretion of apolipoprotein B-containing very low density lipoprotein (86) (**Figure 5**).

### 5.4 Bile acid and nuclear receptor signaling

Consistent with reduced FOXA2 expression in the liver of patients with cholestasis (89, 90), FOXA2-deficient mice exhibited bile acid accumulation and reduced expression of bile acid binding enzyme (Slc27a5), accompanied by transport protein defects due to the fact that FOXA2 directly regulates mrp2 and Oatp2 and indirectly regulates mrp3 and mrp4 (91). The extended list of FOXA2 targets (92) contains high overexpression of the “molecular transporter” category, further supporting the phenotype that FOXA2 deficiency leads to reduced bile acid transporter expression and cholestasis.

Hepatic bile acid accumulation in FOXA2 gene deficient mice induced activation of IRF3 and NF-κB, which regulate inflammatory signaling. Serum lipocalin levels associated with liver inflammation showed an increasing trend and pro-inflammatory protein resistin levels were induced. Furthermore, the acute phase response (APR), the body’s defense response to inflammation, was activated (93).

Analysis of overexpressed functional classes in the genes of FOXA2 mutants suggests that their toxic pathways include liver damage (92), and an experiment also demonstrated increased cholestatic liver injury in cholic acid (CA) diet-fed FOXA2-deficient mice. Cyp3a11 and glutathione S-transferase protect the liver from bile acid toxicity, and FOXA2 deficiency directly resulted in decreased Cyp3a11 expression and indirectly affected glutathione S-transferase expression (91).

The functional class analysis (92) also showed that one of the most common typical pathways among FOXA2 targets is “FXR/RXR activation”. Moreover, the IR-1 response element of FXR is not only present in the promoter of the FOXA2 gene, but is also among the regulatory elements of the direct targets of CA-responsive FOXA2. Jessica Kain’s model (94) has a pioneering ligand-activated binding mechanism:FOXA2 opens chromatin for FXR and LXR binding by expelling nucleosomes during ligand activation, and ligand-dependent activation of FXR and LXR gene expression is also dependent on FOXA2. For example, Cyp3a11, a target of FXR that protects the liver from bile acid toxicity as mentioned above, was increased in ligand activation, whereas Foxa2 mutation altered this increase. However, to ensure ligand-dependent activation of the nuclear receptor, FOXA2 limits the activity of the competing receptor PPAR, and only in FOXA2 mutants, where the PPAR-binding DR-1 element is highly concentrated in regions of increased accessibility and the PPAR target is upregulated.

## 6 Endoplasmic reticulum stress

Endoplasmic reticulum stress can influence hepatic lipid metabolism by inducing *de novo* adipogenesis, interfering with the secretion of lipoproteins and VLDL. In addition to triggering insulin resistance through the accumulation of fat, endoplasmic reticulum stress can affect insulin through each of the unfolded protein response (UPR) pathways (95).

Caizhi Liu et al. (76) demonstrated that FOXA3 was induced by endoplasmic reticulum stress and transcriptionally activated by XBP1 in the unfolded protein response (UPR) pathway by using tunicamycin to induce acute endoplasmic reticulum stress and high-fat diet to induce chronic endoplasmic reticulum stress. FOXA3 further upregulated lipid synthesis gene programs through its target PER1, leading to hepatic steatosis. More importantly, the regulatory axis may also be present in humans.

In mice fed standard chow, mild cholestasis due to the lack of FOXA2 in the liver was sufficient to induce endoplasmic reticulum stress. They showed enhanced levels of the chaperone protein Bip and ubiquitin, a major element of the ubiquitin-proteasome system (UPS), as well as other markers of endoplasmic reticulum stress were also increased in the liver to varying degrees (91). Studies by Thomas S. Weiss et al. suggest that FOXA2 expression is reduced during hepatic steatosis, and FOXA2 regulates its target endogenous ALR level, which may lead to increased endoplasmic reticulum stress and lipid deposition, reduced fatty acid Β-oxidation, ultimately exacerbating the progression of NASH (73, 96). Hepatic progenitors and mature hepatocytes differentiated by FOXA2 mutant induced pluripotent stem cells (FOXA2^-/-^IPSC) exhibited increased expression of endoplasmic reticulum stress markers. Mature hepatocytes showed increased lipid accumulation and overcame lipid accumulation and the resulting toxicity through increased levels of free glycerol and CYP3A4 (97).

## 7 Lipoprotein

FOXA2 binds to the ABCA1 promoter, which inhibits ABCA1 gene expression and LXRα/RXRα-mediated transactivation, possibly thereby regulating intracellular cholesterol and phospholipid efflux to lipid-free ApoA-I and HDL biosynthesis (98). Christian Wolfrum et al. (99) demonstrated that FOXA2 binds to the ApoM promoter and increases plasma HDL levels by regulating ApoM. In addition, ApoM is required for this pathway, because its absence causes the loss of FOXA2’s effect on HDL. ApoaI is a direct target of FOXA3. Hepatic FOXA3 is a positive regulator of plasma ApoA-I, HDL-C levels and reversal of cholesterol transport (100).

## 8 Uncoupling protein

UCP increases energy expenditure and reduces the risk of obesity (101, 102). High-fat diet-induced FOXA2-deficient mice showed increased fat deposition, significantly higher body weight and reduced resting heat and carbon dioxide production than the same diet-induced wild mice, while UCP2 mRNA levels, which can bind to FOXA2 in adipose tissue, were found to be decreased by 50% (79). Xinran Ma et al. demonstrated by FOXA3 knockout, adenovirus injection of FOXA3 and shFOXA3 that the increase or decrease of FOXA3 expression showed an opposite trend to the changes in expression of UCP1 and thermogenic program genes (103). Increased expression of FOXA1 in hepatocytes enhanced UCP1 by 30-35 fold and decreased mitochondrial membrane potential (72).

## 9 Regulation of age-related metabolism

Young mice with hepatic FOXA2 mutation (2-3 months old) had similar body weight compared to the control group. However, In 8- and 11-month-old mice, FOXA2 mutation resulted in higher body weight with elevated fat mass and lean mass. Although obese mice with FOXA2 deficiency eat less, their energy expenditure is also reduced and this performance is also similar to the aged metabolic phenotype. In addition, FOXA2 mutant mice showed reduced growth hormone levels and Stat5b signaling, and gene expression in young Foxa2-deficient mice was similar to that of older male mice, indicating that FOXA2 mutations exhibited a premature aging phenotype. Prior to metabolic changes, cytokine signaling was improperly regulated in young FOXA2-deficient mice. The absence of inhibitory effects of FOXA2 on the MTOR1 pathway, combined with reduced Stat5b signaling, contributed to the induction of Srebp-1c, increased hepatic adipogenesis, and led to age-related obesity (93). Increased occupancy of FOXA2 in the aging liver can de-repress PPAR and LXR-dependent gene expression, leading to the development of steatosis (104).

FOXA3 was directly involved in the development of age-related obesity and insulin resistance (101), and levels of FOXA3 were significantly and selectively increased in brown and inguinal white fat depots during aging. Aging FOXA3 gene deficient mice increased white adipose browning and thermogenesis, reduced adipose tissue expansion, improved hepatic steatosis and insulin sensitivity, and extended lifespan. The mechanism of FOXA3 regulation of the brown fat gene program involves inhibition of PGC1α levels by interfering with cAMP response element binding protein 1-mediated transcriptional regulation of the PGC1α promoter.

## 10 Conclusions

**Figure 6:**
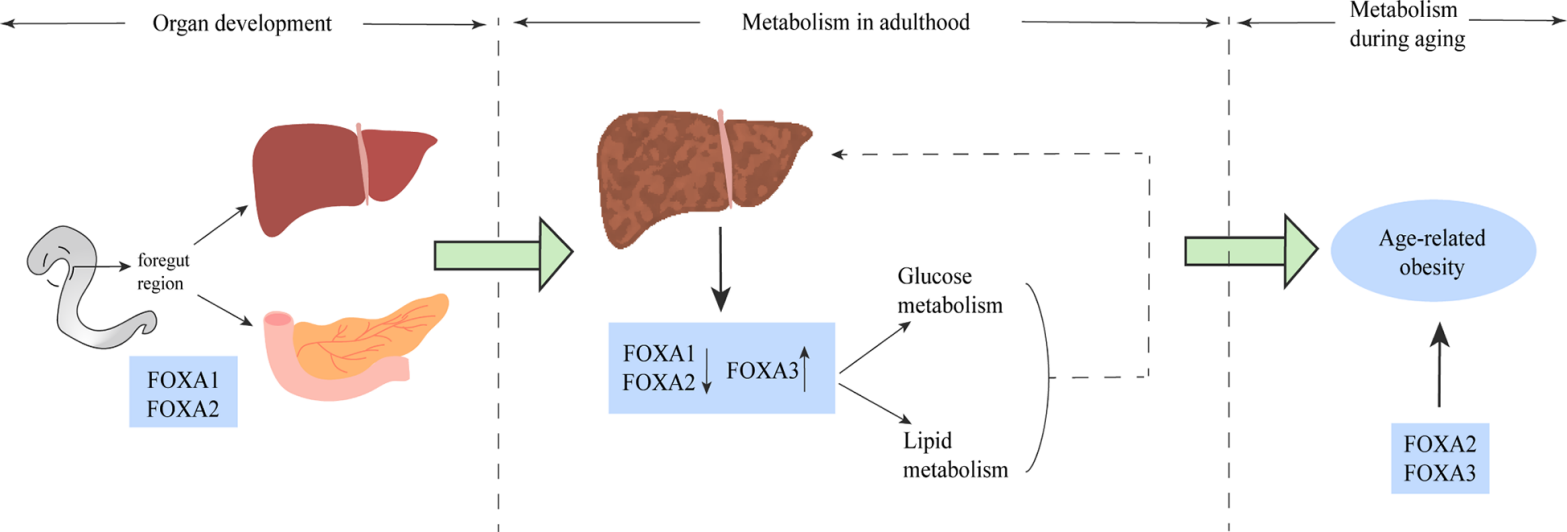
The FOXA family plays a role in multiple stages of life. FOXA1 and FOXA2 play a major role in the phase of liver and spleen development. The role of the FOXA family for glucose metabolism and lipid metabolism is well established (solid line), and altered FOXA expression has been shown in the liver of both patients and animal models of NAFLD, and some of these alterations have been proven to have impact on the pathogenesis of NAFLD (dashed line). In older age, mainly FOXA2 and FOXA3 dominate the development of age-related obesity.

Based on the above findings, the FOXA protein family plays an important role in multiple stages of mammalian life, from embryonic development to glucolipid metabolism, hepatic steatosis and the development of NAFLD, to age-related obesity. The effects of FOXA on metabolism and development appear to be important and profound (**Figure 6**). Moreover, the effects of FOXA on glucose and lipid metabolism are multifaceted, the relevant studies are summarized in **Table 2**. In glucose metabolism, FOXA is necessary for α and Β cell maturation and related secretory functions, and has regulatory effects on glucagon gene promoter, GLP-1, hepatic gluconeogenesis and glycolysis. In lipid metabolism, FOXA also has effects on important metabolic processes such as fatty acid and bile acid metabolism, etc.

**Table 2:** Contribution of forkhead box (FOX) protein to glycolipid metabolism.

FOXA members	Model	Main results	Ref
FOXA1	null mutation in FOXA1	1. Severe postnatal growth retardation, early postnatal mortality 2. Hypoglycemic, plasma glucagon and insulin levels are very low. Corticosteroid levels are very high	(25, 48)
	Isolated Foxa1-/- islet	1. Reduced glucose-stimulated insulin release, total pancreatic insulin, glucagon content and insulin mRNA level↓ 2. Intracellular Ca2^+^ and ATP levels↓ 3. mRNA level of Ucp-2↑	(37)
	STC-1 cells were transfected with miR-194 inhibitor+si-Foax1	The active GLP-1 level, mRNA levels of gcg and pcsk1, the protein level of PC1/3↓	(56)
	HepG2 cells infected with FoxA1 shRNA	mRNA levels of IGFBP1 and G6Pase↓	(60)
	Human hepatocytes and HepG2 cells transfected with ad-Foax1	1. liver lipid accumulation↓, TG synthesis-related gene: mRNA levels of GPAT1, DGAT2↓ 2.VLDL synthesis-related gene: mRNA levels of DGAT2, MTP, ACAT2, APOB↓ 3 Fatty acid transporter protein: mRNA level of FATP2↓and fatty acid beta oxidation: mRNA levels of CROT, Acox↑ 4. Ketone content and ketogenesis genes: mRNA level of: HMGCS2↑ 5. mRNA levels of UCP1and MMP↓	(72)
	NAFLD patients or MCD rats	mRNA level of FOXA1↓	(72)
	3T3-L1 cells transfected with FOXA1 siRNA	lipid accumulation↑, adipogenic genes: mRNA levels of PPARγ, aP2 and SCD↑	(78)
	HepG2 treated with Oleic acid and GLP-1R agonist exendin-4	mRNA levels of FOXA1 and FABP1↓	(105)
FOXA2	Infants with FOXA2 gene mutation	Congenital hyperinsulinism and hypoglycemia	(39)
	Β cell specific Foxa2 deletion	1.Hyperinsulinemic hypoglycemia: blood glucose levels and GTT↓,plasma insulin and glucagon levels↑ 2. mRNA levels of Kir6.2, Sur1, Kcnb1, Ffar2, Hadhsc, Ddc, Pdx1, SHP↓, Glut5, DCK, PDK1,HNF4α, PGC1α, CREB, Nrip1, Ch.B, Prka1a, VAPB, Mtac2, Rasgrp1, Myh10↑	(40)
	Pancreatic Βcell–specific Foxa2 knockout mice (Foxa2^loxP/loxP^; Ins.Cre)	1.Postnatal growth retardation, early postnatal mortality 2.Hyperinsulinemic hypoglycemia and elevated liver glycogen content 3. Kir6.2 and SUR1 mRNA levels in islet↓	(41)
	MIN6 pseudoislets + miR141 + FOXA2/INS-1 cells+miR-141 + FOXA2	Glucose-stimulated insulin secretion↓pancreatic Β cell proliferation↑	(42)
	FOXA2^+/-^ induced pluripotent stem cells-derived pancreatic progenitor (PP2)/endocrine progenitor (EP)/Β cell	1.mRNA levels of PDX1, Nkx6.1 SOX9, GATA6, ONecut1, HNF1A, PAX4, PTF1 and AMYLASE/NGN3,CHGA,NEUROD1,NKX6.1, and NKX2.2/INS, Nkx6.1, C-PEP, GCG, ABCC8, KCNJ11, NKX2.2and PDX1↓ 2. The total content of insulin and number of Β cells↓	(43)
	Endoderm-specific Foxa2 knockout mice (Foxa2^loxP/loxP^; Foxa3Cre)	1.Early lethality and severe hypoglycemia 2.Islets displayed a disorganized architecture, few mature α-cells and decreased plasma glucagon	(47)
	mouse primary hepatocyte infected with FoxA2 shRNA	Glucose production, mRNA levels of Shp/Nr0b2↓	(63)
	Fasted hepatocyte FOXA2 knockout mice (Foxa2^loxP/loxP^; AlfpCre)/Primary hepatocytes were isolated Foxa2^loxP/loxP^; AlfpCre mice and exposed to glucagon or glucocorticoid	Hepatic mRNA levels of PEPCK、IGFBP1 and Tat↓	(64)
	Mice tail vein injections of adenovirus expressing the FOXA2(Ad-FOXA2)	1. moderate hepatocyte damage 2.Diminished hepatic Glycogen Storage, transient decrease in fasting blood glucose levels 3.Hepatic mRNA levels of PEPCK, G6P, Glut2, Ntcp, Spgp and UDPGT↓ 4. Serum levels of bile acid↑	(65)
	T-77 transgenic mouse	1.Diminished hepatic Glycogen Storage and serum levels of glucose 2. Transient substantial hepatic steatosis, hepatic mRNA levels of fatty acid synthase: GPAT, AOX, L-PBE, asparagine synthase and glutamate dehydrogenase↑	(66)
	SREBP-1c mice injected with Ad-FOXA2T156A	1. Body weight, liver triglyceride levels, blood glucose concentrations and plasma insulin↓, plasma triglycerides, free fatty acids and ketone bodies↑ 2. Resting O2 consumption, CO2 production and heat production↑ 3. Hepatic mRNA levels of G6PC, PPARγ, UCP-2, UCP-3, Irs2,p-Akt↑, Fas and SCD-1↓	(67)
	Haploinsufficient Foxa2 (Foxa2+/-) mice fed a high-fat diet	Production of CO2 and ketone body generation↓,Plasma triglyceride, free fatty acid and glucose output↑	(67)
	hepatic progenitors cell transfected with FoxA2 shRNA	1.Ki‐67 positive rate and the expression of PCNA↑ 2.Higher glycolysis, glycolytic capacity, and glycolytic reserve capacity, the mRNA, protein expression of HK2 and HK2 activity↑	(68)
	patients with hepatic steatosis and NASH	mRNA level of hepatic FOXA2↓	(73)
	HFD-fed mice and diabetic fa/fa rats	mRNA level of hepatic FOXA2↑	(74)
	Huh7 cells infected with FOXA2-siRNA	mRNA levels of HMGCS2, ABHD5 and PPARGC1A↓	(74)
	primary preadipocytes of Foxa-2^+/–^	mRNA levels of pref-1↓, aP2↑	(79)
	FOXA-2+/- mice fed a high-fat diet	1.Weight gain↑, pericardial, intraperitoneal, and subcutaneous fat deposits↑ 2. Resting heat and CO2 production↓ 3. mRNA levels of Foxa-2, Glut-4, Hk-2, M2Pk, Irs-2, Ucp-2, and Ucp-3 in adipocytes↓	(79)
	HepG2 cells were transfected with FOXA2 siRNA	mRNA and protein levels of LPL↓	(82, 83)
	HepG2 cells were treated with insulin	Protein levels of nuclear FOXA2↓cytoplasmic FOXA2↑, pAKT↑, mRNA level of LPL↓	(85)
	Obese db/db mice were injected with recombinant adenovirus expressing Foxa2 mutants(Foxa2 K259Q, Foxa2 T156A, Foxa2 T156A-K259Q)	1.Plasma insulin levels, blood glucose levels, liver triglyceride↓, 2.Hepatic and plasma Β-oxidation and ketone body production↑	(85)
	ob/ob mice were injected with an adenovirus expressing Foxa2T156A and Pgc-1Β	1. Liver weight, liver TG↓, plasma TG, plasma FFA concentrations↑ 2.Mitochondrial Β oxidation and genes involved in Β oxidation: Mcad and Vlca↑ 3.Plasma glucose and insulin levels↓ 4. Genes involved in hepatic VLDL secretion: mRNA levels of Mtp, Dgat2↑	(86)
	Huh7.5cells infected with HCV and FoxA2 plasmid-transfected	1. HCV virus replication and lipid droplet formation↓ 2. Key enzymes involved in Β-oxidation:protein levels of MCAD and SCAD↑	(87)
	Primary human hepatocytes were isolated from cholestatic liver tissues	mRNA level of FOXA2↓	(89)
	primary sclerosing cholangitis and primary biliary cholangitis patients	Hepatic mRNA level of FOXA2↓	(90)
	Foxa2 mutant mice (Foxa2^loxP/loxP^ Alfp.Cre) fed on a diet containing cholic acid	1. Bile acids, ALT, AST↑ 2. Genes involved in bile formation and hepatic bile acid transport:hepatic mRNA and protein levels of Mrp2, Mrp3, Mrp4, Oatp2, Cyp3a11↓ 3. ER stress: ER dilation, BIP and cytoplasmic ubiquitin↑ 4. Hepatic glutathione enzymes: hepatic mRNA levels of Gsta1, Gsta2, Gstm2↓	(91)
	Young Foxa2-deficient mice (Foxa2^loxP/loxP^ Alfp.Cre mice)	1. The acute phase response (APR): Serum levels of acute phase reactants alpha-2-Macroglobulin (A2M), C-reactive protein (CRP)↑ and High-density lipoprotein (HDL)cholesterol levels↓ 2. Circulating levels of growth hormone (GH)↓and resistin↑	(93)
	Old Foxa2-deficient mice (Foxa2^loxP/loxP^ Alfp.Cre mice)	1. Weight gain↑, food and water intake↓, and energy expenditure (oxygen consumed and carbon dioxide exhaled)↓ 2. Hepatic mRNA levels of Srebp1-c, Fasn↑ Pgc1α↓ 3. The primary substrate of mTORC1: hepatic protein levels of p-s6k, p-4E-BP1↑and repressors of mTOR : Ddit4, SESN3, Trib3 and TSC1↓ 4. Circulating levels of GH↓	(93)
	hepatic progenitors or mature hepatocytes derived fromFOXA2^−/−^iPSCs	1. ER stress genes:mRNA levels of ATF4,CHOP,EDEM,PUMA,XBP1s,DNAJBP,DP5, and IL6↑ 2. Albumin concertation↓ 3. The levels of glycerol and CYP3A4↑ 4. glucose uptake and glycogen accumulation↓	(97)
	HepG2 cells were treated with adenovirus of FOXA2	mRNA level of ABCA1↓	(98)
	Wild-type C57Bl/6, ob/ob, and HF-fed mice that injected with Ad- FOXA2 T156A	Protein level of apoM in the liver and plasma↑, circulating cholesterol levels↑and preΒ-HDL levels↑	(99)
	Foxa2^+/-^ mice	Protein levels of apoM in the liver and plasma↓and plasma HDL cholesterol levels↓	(99)
	primary hepatocytes infected with adenovirus of FOXA2	Hepatic protein levels of Ntcp and Cyp7a1↑	(106)
	IRT1-mutant mice were injected with adenovirus of FOXA2	1.Levels of bile acid↑/cholesterol and triglyceride↓in serum and liver 2. Hepatic mRNA levels of genes involved in bile acid synthesis and transport: Cyp7a1, Cyp8b1, Cyp7b1, Slc10A1, Abcc2, Shp,CPT-1a↑, Abcc3↓	(106)
FOXA3	Foxa3 null mice fasting for 24-72 hours	1.lower blood glucose levels 2. Hepatic mRNA levels of PEPCK、G6Pase and Tat↑, Glut2↓	(71)
	FOXA3 KO mice fed on HFD for 12 weeks	1. liver weight, liver TG levels, GTT,ITT↓ 2. Lipogenesis gene: mRNA levels of PPARγ, Srebp1c, Fasn, Scd1, Acc2, Gpat2↓ 3. Inflammation gene: mRNA levels of Tnfα, IFNγ, ll1Β, ll12p40, Cox2, Mcp1↓ 4. Endoplasmic reticulum stress gene: mRNA levels of Bip, Chop↓	(76)
	10T1/2 cells infected with Foxa3-siRNA	mRNA levels of adipocyte markers: aP2, PPARγ, C/EBPα, -Β, and -δ, perilipin, and adiponectin↓	(80)
	Foxa3-null mice + HFD	1. Body weight, Weights of epididymal, Size measurements of adipocytes in the epididymal↓ 2. GTT, ITT↓, serum adiponectin↑, insulin and cholesterol levels↓ 3. mRNA levels of adipocyte differentiation markers in epididymal fat depots: PPARγ, aP2, and adiponectin↓	(80)
	ob/ob mice, db/db mice, HFD-fed mice and patients with NASH	Hepatic mRNA or proteins levels of FOXA3↓	(100)
	C57BL/6J mice were injected with Ad-shFoxa3	1.Plasma HDL-C↓, ALT and AST levels↑ 2.mRNA levels of hepatic genes involved in lipoprotein uptake(Apoe), HDL biogenesis(Apoa1, Apoa2, Apom), or inflammation(Pon1)↓(Il-6, Tnf-α, Saa1, Saa2, Saa3)↑	(100)
	Foxa3-null (Foxa3 KO) mice	1.Survival%↑ 2.Body weights, body fat and adipose tissue weights(BAT, eWAT, iWAT rWAT, mWAT↓ 3. Liver TG, GTT, ITT and serum leptin and cholesterol levels↓ 4. Oxygen consumption at room temperature (24°C) and body temperatures exposed to cold (4°C)↑ 5. mRNA levels of thermogenic and mitochondrial genes in BAT and iWAT: Pgc1α, Ucp1, CytC, Mcad, Cox4Β, Atpase↑	(103)
FOXA1/2	Compound ablation of Foxa1 and Foxa2 in Β-cells (Foxa1^loxP/loxP^; Foxa2^loxP/loxP^; Pdx1CreERT2)	1. Severe hypoglycemia, hyperinsulinemia and impaired Ca2+ oscillation 2. ChREBP protein and mRNA levels↓	(44)
	siRNA directed against Foxa1 and Foxa2 in rat primary pancreaticα-cells and rodent α-cell lines	1.Glucagon content↓ 2. α cell differentiation gene: MafB, Pou3f4, Nkx2.2↓, glucagon synthesis and secretion gene: Pcsk2, Sur1, glucagon, Isl1, Kir6.2, Sur1, GIPR↓	(46)
	Intestine-Specific Deletion of Foxa1 and Foxa2	1. The growth rate↓ 2. Goblet cell numbers and the mucin glycoproteins↓, partially or completely empty vacuoles 3. Glp-1–, Glp-2–positive cells are undetectable 4. mRNA levels of preproglucagon, ISL-1 and Pax6↓	(55)
FoxA1/2/3	FoxA1^L/L^/FoxA2^L/L^/FoxA3^-/-^ mice (FoxA triple null)	1. Weight loss started after 15 days and 20% of the mice died within the next 4 days 2. Significant elevation of plasma ALT, ALKP and severe liver damage 3.Hyperargininemia, hypouremia, and hyperammonemia,	(32)

Although the results of some studies on the role of FOXA appear to be relative:the glucagon-like peptide-1 receptor (GLP-1R) agonist Exendin-4 leads to reduced expression of FOXA1 *via* the Wnt/Β-catenin pathway, further resulting in a decrease in FABP1, which causes a reduction in fatty acid uptake and prevents the development of hepatic steatosis (105), which seems contradictory to the reduction in fatty acid uptake by FOXA1 found in *in vitro* experiments. In addition to the difference in the trend of FOXA2 expression in liver tissue from patients with hepatic steatosis and animal models, Qi Chen et al. found that metformin treatment induced intrahepatic cholestasis and liver injury in a minority number of type II diabetic patients possibly through inhibition of SIRT1 leading to elevated FOXA2 acetylation and transcriptional activity. Thus, promoting the binding of FOXA2 and promoters of bile acid synthesis and transport-related genes such as CYP7A1, NTCP and the expression of these genes (106), which is different from the effect of FOXA2 on the prevention of bile acid accumulation and associated inflammation and liver injury mentioned above. Changes in FOXA3 expression in NAFLD patients and animal models remain to be further determined.

Despite the fact that many biological effects of the FOXA protein family on glucose and lipid metabolism have been demonstrated in animal and cellular models, only some of these pathways have been directly shown to cause NAFLD, and the corresponding clinical trials are focused on breast cancer (107), prostate cancer (108) and other cancers. There are still few clinical trials on glycolipid metabolism and other human studies are mostly based on induced pluripotent stem cells *in vitro* as models, so more room for research is still available. Moreover, the three members of the FOXA family, FOXA1, FOXA2, and FOXA3, have high structural homology, each of which binds closely to either of the other two on the same DNA molecule in the same hepatocyte, and some of them even interact with each other at the binding site (109). In particular, their roles are redundant in many regulatory processes of glycolipid metabolism. Therefore, whether one or more of these members can be targeted for the treatment or prevention of glucolipid metabolism-related diseases such as NAFLD is something that needs to be further investigated and explored.

## Author contributions

All authors listed have made a substantial, direct, and intellectual contribution to the work, and approved it for publication.

## References

[1] Bechmann LP Hannivoort RA Gerken G Hotamisligil GS Trauner M Canbay A. The interaction of hepatic lipid and glucose metabolism in liver diseases. J Hepatol (2012) 56(4):952–64. doi: 10.1016/j.jhep.2011.08.025 22173168

[2] Younossi Z Tacke F Arrese M Chander Sharma B Mostafa I Bugianesi E. Global perspectives on nonalcoholic fatty liver disease and nonalcoholic steatohepatitis. Hepatology (2019) 69(6):2672–82. doi: 10.1002/hep.30251 30179269

[3] Powell EE Wong VW-S Rinella M. Non-alcoholic fatty liver disease. Lancet (2021) 397(10290):2212–24. doi: 10.1016/s0140-6736(20)32511-3 33894145

[4] Loomba R Friedman SL Shulman GI. Mechanisms and disease consequences of nonalcoholic fatty liver disease. Cell (2021) 184(10):2537–64. doi: 10.1016/j.cell.2021.04.015 PMC12168897 33989548

[5] Friedman SL Neuschwander-Tetri BA Rinella M Sanyal AJ. Mechanisms of nafld development and therapeutic strategies. Nat Med (2018) 24(7):908–22. doi: 10.1038/s41591-018-0104-9 PMC6553468 29967350

[6] Jiao N Baker SS Chapa-Rodriguez A Liu W Nugent CA Tsompana M. Suppressed hepatic bile acid signalling despite elevated production of primary and secondary bile acids in nafld. Gut (2018) 67(10):1881–91. doi: 10.1136/gutjnl-2017-314307 28774887

[7] Targher G Corey KE Byrne CD Roden M. The complex link between nafld and type 2 diabetes mellitus - mechanisms and treatments. Nat Rev Gastroenterol Hepatol (2021) 18(9):599–612. doi: 10.1038/s41575-021-00448-y 33972770

[8] Lai E PV Tao WF Chen WS Darnell JE Jr. Hepatocyte nuclear factor 3 alpha belongs to a gene family in mammals that is homologous to the drosophila homeotic gene fork head. Genes Dev (1991) 5(3):416–27. doi: 10.1101/gad.5.3.416 1672118

[9] Hannenhalli S Kaestner KH. The evolution of fox genes and their role in development and disease. Nat Rev Genet (2009) 10(4):233–40. doi: 10.1038/nrg2523 PMC2733165 19274050

[10] Zaret KS Carroll JS. Pioneer transcription factors: Establishing competence for gene expression. Genes Dev (2011) 25(21):2227–41. doi: 10.1101/gad.176826.111 PMC3219227 22056668

[11] Heslop JA Duncan SA. Foxa factors: The chromatin key and doorstop essential for liver development and function. Genes Dev (2020) 34(15-16):1003–4. doi: 10.1101/gad.340570.120 PMC7397850 32747476

[12] Zaret KS. Pioneer transcription factors initiating gene network changes. Annu Rev Genet (2020) 54:367–85. doi: 10.1146/annurev-genet-030220-015007 PMC7900943 32886547

[13] Fernandez Garcia M Moore CD Schulz KN Alberto O Donague G Harrison MM. Structural features of transcription factors associating with nucleosome binding. Mol Cell (2019) 75(5):921–32e6. doi: 10.1016/j.molcel.2019.06.009 31303471 PMC6731145

[14] Lisa A.Cirillo CEM Pascale Bossard KS Sindhu Cherian EYS SKBa KL S.Zaret K. Binding of the winged-helix transcription factor Hnf3 to a linker histone site on the nucleosome. EMBO J (1998) 17:244–54. doi: 10.1093/emboj/17.1.244 PMC1170375 9427758

[15] Taube JH Allton K Duncan SA Shen L Barton MC. Foxa1 functions as a pioneer transcription factor at transposable elements to activate afp during differentiation of embryonic stem cells. J Biol Chem (2010) 285(21):16135–44. doi: 10.1074/jbc.M109.088096 PMC2871482 20348100

[16] Balsalobre A Drouin J. Pioneer factors as master regulators of the epigenome and cell fate. Nat Rev Mol Cell Biol (2022) 23(7):449–64. doi: 10.1038/s41580-022-00464-z 35264768

[17] Wang W Yao LJ Shen W Ding K Shi PM Chen F. Foxa2 alleviates Ccl4-induced liver fibrosis by protecting hepatocytes in mice. Sci Rep (2017) 7(1):15532. doi: 10.1038/s41598-017-15831-6 29138513 PMC5686201

[18] Gao B Xie W Wu X Wang L Guo J. Functionally analyzing the important roles of hepatocyte nuclear factor 3 (Foxa) in tumorigenesis. Biochim Biophys Acta Rev Cancer (2020) 1873(2):188365. doi: 10.1016/j.bbcan.2020.188365 32325165

[19] Lai E PV Smith E Litvin O Costa RH Darnell JE Jr. Hnf-3a, a hepatocyte-enriched transcription factor of novel structure is regulated transcriptionally. Genes Dev (1990) 4(8):1427–36. doi: 10.1101/gad.4.8.1427 2227418

[20] Kaestner KH KW Martinez DE. Unified nomenclature for the winged Helix/Forkhead transcription factors. Genes Dev (2000) 15;14(2):142–6. doi: 10.1101/gad.14.2.142 10702024

[21] Friedman JR Kaestner KH. The foxa family of transcription factors in development and metabolism. Cell Mol Life Sci (2006) 63(19-20):2317–28. doi: 10.1007/s00018-006-6095-6 PMC11136376 16909212

[22] Tachmatzidi EC Galanopoulou O Talianidis I. Transcription control of liver development. Cells (2021) 10(8):2026. doi: 10.3390/cells10082026 34440795 PMC8391549

[23] Lau HH Ng NHJ Loo LSW Jasmen JB Teo AKK. The molecular functions of hepatocyte nuclear factors - in and beyond the liver. J Hepatol (2018) 68(5):1033–48. doi: 10.1016/j.jhep.2017.11.026 29175243

[24] Clark KL HE Lai E Burley SK. Co-Crystal structure of the hnf-3/Fork head DNA-recognition motif resembles histone H5. Nature (1993) 364(6436):412–20. doi: 10.1038/364412a0 8332212

[25] Kaestner KH KJ Liu Y Drucker DJ Schütz G. Inactivation of the winged helix transcription factor Hnf3alpha affects glucose homeostasis and islet glucagon gene expression in vivo. Genes Dev (1999) 13(4):495–504. doi: 10.1101/gad.13.4.495 10049364 PMC316473

[26] Kaestner KH HH Schütz G. Targeted disruption of the gene encoding hepatocyte nuclear factor 3gamma results in reduced transcription of hepatocyte-specific genes. Mol Cell Biol (1998) 18(7):4245–51. doi: 10.1128/MCB.18.7.4245 PMC109008 9632808

[27] Lee CS Friedman JR Fulmer JT Kaestner KH. The initiation of liver development is dependent on foxa transcription factors. Nature (2005) 435(7044):944–7. doi: 10.1038/nature03649 15959514

[28] Kaestner KH. The making of the liver: Developmental competence in foregut endoderm and induction of the hepatogenic program. Cell Cycle (2005) 4(9):1146–8. doi: 10.4161/cc.4.9.2033 16123587

[29] Si-Tayeb K Lemaigre FP Duncan SA. Organogenesis and development of the liver. Dev Cell (2010) 18(2):175–89. doi: 10.1016/j.devcel.2010.01.011 20159590

[30] Genga RMJ Kernfeld EM Parsi KM Parsons TJ Ziller MJ Maehr R. Single-cell rna-Sequencing-Based crispri screening resolves molecular drivers of early human endoderm development. Cell Rep (2019) 27(3):708–18e10. doi: 10.1016/j.celrep.2019.03.076 30995470 PMC6525305

[31] Strazzabosco Mario. Foxa1 and Foxa2 regulate bile duct development in mice. J Hepatol (2010) 52(5):765–7. doi: 10.1016/j.jhep.2009.12.022 PMC2862815 20347503

[32] Reizel Y Morgan A Gao L Lan Y Manduchi E Waite EL. Collapse of the hepatic gene regulatory network in the absence of foxa factors. Genes Dev (2020) 34(15-16):1039–50. doi: 10.1101/gad.337691.120 PMC7397852 32561546

[33] Thakur A Wong JCH Wang EY Lotto J Kim D Cheng JC. Hepatocyte nuclear factor 4-alpha is essential for the active epigenetic state at enhancers in mouse liver. Hepatology (2019) 70(4):1360–76. doi: 10.1002/hep.30631 PMC6773525 30933372

[34] Kaestner KH. The hepatocyte nuclear factor 3 (Hnf3 or foxa) family in metabolism. Trends Endocrinol Metab (2000) 11(7):281–5. doi: 10.1016/s1043-2760(00)00271-x 10920385

[35] Lee K CH Rickert RW Li QV Pulecio J Leslie CS Huangfu D. Foxa2 is required for enhancer priming during pancreatic differentiation. Cell Rep (2019) 28(2):382–93. doi: 10.1016/j.celrep.2019.06.034 PMC6636862 31291575

[36] Gao N LJ Vatamaniuk MZ Rieck S Friedman JR Kaestner KH. Dynamic regulation of Pdx1 enhancers by Foxa1 and Foxa2 is essential for pancreas development. Genes Dev (2008) 22(24):3435–48. doi: 10.1101/gad.1752608 PMC2607077 19141476

[37] Vatamaniuk MZ GR Lantz KA Doliba NM Matschinsky FM Kaestner KH. Foxa1-deficient mice exhibit impaired insulin secretion due to uncoupled oxidative phosphorylation. Diabetes (2006) 55(10):2730–6. doi: 10.2337/db05-0470 17003337

[38] Theis A SR Garofalo D Paul A Narayana A Sussel L. Groucho Co-repressor proteins regulate Β cell development and proliferation by repressing Foxa1 in the developing mouse pancreas. Development (2021) 148(6):dev192401. doi: 10.1242/dev.192401 33658226 PMC8015241

[39] Vajravelu ME CJ Krock B Baker S Langdon D Alter C De León DD. Congenital hyperinsulinism and hypopituitarism attributable to a mutation in Foxa2. J Clin Endocrinol Metab (2018) 103(3):1042–7. doi: 10.1210/jc.2017-02157 PMC6276717 29329447

[40] Gao N WP Doliba N Golson ML Matschinsky FM Kaestner KH. Foxa2 controls vesicle docking and insulin secretion in mature beta cells. Cell Metab (2007) 6(4):267–79. doi: 10.1016/j.cmet.2007.08.015 17908556

[41] Sund NJ VM Casey M Ang SL Magnuson MA Stoffers DA Matschinsky FM. Tissue-specific deletion of Foxa2 in pancreatic beta cells results in hyperinsulinemic hypoglycemia. Genes Dev (2001) 15(13):1706–15. doi: 10.1101/gad.901601 PMC312732 11445544

[42] Yu X Zhong L. Pioglitazone/Microrna141/Foxa2: A novel axis in pancreatic betacells proliferation and insulin secretion. Mol Med Rep (2018) 17(6):7931–8. doi: 10.3892/mmr.2018.8813 29620270

[43] Elsayed AK Younis I Ali G Hussain K Abdelalim EM. Aberrant development of pancreatic beta cells derived from human ipscs with Foxa2 deficiency. Cell Death Dis (2021) 12(1):103. doi: 10.1038/s41419-021-03390-8 33473118 PMC7817686

[44] Gao N Le Lay J Qin W Doliba N Schug J Fox AJ. Foxa1 and Foxa2 maintain the metabolic and secretory features of the mature beta-cell. Mol Endocrinol (2010) 24(8):1594–604. doi: 10.1210/me.2009-0513 PMC2940470 20534694

[45] Campbell JE Newgard CB. Mechanisms controlling pancreatic islet cell function in insulin secretion. Nat Rev Mol Cell Biol (2021) 22(2):142–58. doi: 10.1038/s41580-020-00317-7 PMC8115730 33398164

[46] Heddad Masson M Poisson C Guerardel A Mamin A Philippe J Gosmain Y. Foxa1 and Foxa2 regulate alpha-cell differentiation, glucagon biosynthesis, and secretion. Endocrinology (2014) 155(10):3781–92. doi: 10.1210/en.2013-1843 25057789

[47] Lee CS Sund NJ Behr R Herrera PL Kaestner KH. Foxa2 is required for the differentiation of pancreatic alpha-cells. Dev Biol (2005) 278(2):484–95. doi: 10.1016/j.ydbio.2004.10.012 15680365

[48] Shih DQ NM Kuwajima S Duncan SA Stoffel M. Impaired glucose homeostasis and neonatal mortality in hepatocyte nuclear factor 3alpha-deficient mice. Proc Natl Acad Sci U.S.A. (1999) 96(18):10152–7. doi: 10.1073/pnas.96.18.10152 PMC17858 10468578

[49] Sharma SK LU Ratke R Oetjen E Blume R Dickel C Knepel W. Characterization of a novel foxa (Hepatocyte nuclear factor-3) site in the glucagon promoter that is conserved between rodents and humans. Biochem J (2005) 389(Pt 3):831–41. doi: 10.1042/BJ20050334 PMC1180734 15828872

[50] Liu Y SW Brubaker PL Kaestner KH Drucker DJ. Foxa3 (Hnf-3gamma) binds to and activates the rat proglucagon gene promoter but is not essential for proglucagon gene expression. Biochem J (2002) 366(Pt 2):633–41. doi: 10.1042/BJ20020095 PMC1222783 12000309

[51] Philippe J MC Prezioso VR. Glucagon gene expression is negatively regulated by hepatocyte nuclear factor 3 beta. Mol Cell Biol (1994) 14(5):3514–23. doi: 10.1128/mcb.14.5.3514-3523.1994 PMC358715 8164696

[52] Gauthier BR SV Zaiko M Mamin A Ritz-Laser B Philippe J. Hepatic nuclear factor-3 (Hnf-3 or Foxa2) regulates glucagon gene transcription by binding to the G1 and G2 promoter elements. Mol Endocrinol (2002) 16(1):170–83. doi: 10.1210/mend.16.1.0752 11773447

[53] Holst JJ. The physiology of glucagon-like peptide 1. Physiol Rev (2007) 87(4):1409–39. doi: 10.1152/physrev.00034.2006 17928588

[54] Drucker DJ. Mechanisms of action and therapeutic application of glucagon-like peptide-1. Cell Metab (2018) 27(4):740–56. doi: 10.1016/j.cmet.2018.03.001 29617641

[55] Ye DZ Kaestner KH. Foxa1 and Foxa2 control the differentiation of goblet and enteroendocrine l- and d-cells in mice. Gastroenterology (2009) 137(6):2052–62. doi: 10.1053/j.gastro.2009.08.059 PMC2789913 19737569

[56] Wang J Zhao D Ding CZ Guo F Wu LN Huang FJ. Microrna-194: A novel regulator of glucagon-like peptide-1 synthesis in intestinal l cells. Cell Death Dis (2021) 12(1):113. doi: 10.1038/s41419-020-03366-0 33479193 PMC7820456

[57] Wakil SJ Abu-Elheiga LA. Fatty acid metabolism: Target for metabolic syndrome. J Lipid Res (2009) 50 Suppl:S138–43. doi: 10.1194/jlr.R800079-JLR200 PMC2674721 19047759

[58] Alves-Bezerra M Cohen DE. Triglyceride metabolism in the liver. Compr Physiol (2017) 8(1):1–8. doi: 10.1002/cphy.c170012 29357123 PMC6376873

[59] Oh KJ Han HS Kim MJ Koo SH. Transcriptional regulators of hepatic gluconeogenesis. Arch Pharm Res (2013) 36(2):189–200. doi: 10.1007/s12272-013-0018-5 23361586

[60] Yalley A Schill D Hatta M Johnson N Cirillo LA. Loss of interdependent binding by the Foxo1 and Foxa1/A2 forkhead transcription factors culminates in perturbation of active chromatin marks and binding of transcriptional regulators at insulin-sensitive genes. J Biol Chem (2016) 291(16):8848–61. doi: 10.1074/jbc.M115.677583 PMC4861452 26929406

[61] Schill D NJ Cirillo LA. Foxo1 and Foxa1/2 form a complex on DNA and cooperate to open chromatin at insulin-regulated genes. Biochem Cell Biol (2019) 97(2):118–29. doi: 10.1139/bcb-2018-0104 30142277

[62] Da L Cao T Sun X Jin S Di X Huang X. Hepatic Tet3 contributes to type-2 diabetes by inducing the Hnf4alpha fetal isoform. Nat Commun (2020) 11(1):342. doi: 10.1038/s41467-019-14185-z 31953394 PMC6969024

[63] Ploton M Mazuy C Gheeraert C Dubois V Berthier A Dubois-Chevalier J. The nuclear bile acid receptor fxr is a pka- and Foxa2-sensitive activator of fasting hepatic gluconeogenesis. J Hepatol (2018) 69(5):1099–109. doi: 10.1016/j.jhep.2018.06.022 29981427

[64] Zhang L Rubins NE Ahima RS Greenbaum LE Kaestner KH. Foxa2 integrates the transcriptional response of the hepatocyte to fasting. Cell Metab (2005) 2(2):141–8. doi: 10.1016/j.cmet.2005.07.002 16098831

[65] Tan Y Hughes D Wang X Costa RH. Adenovirus-mediated increase in hnf-3beta or hnf-3alpha shows differences in levels of liver glycogen and gene expression. Hepatology (2002) 35(1):30–9. doi: 10.1053/jhep.2002.30317 11786957

[66] Hughes DE Stolz DB Yu S Tan Y Reddy JK Watkins SC. Elevated hepatocyte levels of the forkhead box A2 (Hnf-3beta) transcription factor cause postnatal steatosis and mitochondrial damage. Hepatology (2003) 37(6):1414–24. doi: 10.1053/jhep.2003.50253 12774021

[67] Wolfrum C AE Luca E Friedman JM Stoffel M. Foxa2 regulates lipid metabolism and ketogenesis in the liver during fasting and in diabetes. Nature (2004) 432(7020):1027–32. doi: 10.1038/nature03047 15616563

[68] Wang P Cong M Liu T Li Y Liu L Sun S. Foxa2 inhibits the proliferation of hepatic progenitor cells by reducing Pi3k/Akt/Hk2-mediated glycolysis. J Cell Physiol (2020) 235(12):9524–37. doi: 10.1002/jcp.29759 32495363

[69] Lin B MD Chou JY. The role of Hnf1alpha, Hnf3gamma, and cyclic amp in glucose-6-Phosphatase gene activation. Biochemistry. (1997) 36(46):14096–106. doi: 10.1021/bi9703249 9369482

[70] Cha JY Kim H Kim KS Hur MW Ahn Y. Identification of transacting factors responsible for the tissue-specific expression of human glucose transporter type 2 isoform gene. cooperative role of hepatocyte nuclear factors 1alpha and 3beta. J Biol Chem (2000) 275(24):18358–65. doi: 10.1074/jbc.M909536199 10748140

[71] Shen W Scearce LM Brestelli JE Sund NJ Kaestner KH. Foxa3 (Hepatocyte nuclear factor 3gamma ) is required for the regulation of hepatic Glut2 expression and the maintenance of glucose homeostasis during a prolonged fast. J Biol Chem (2001) 276(46):42812–7. doi: 10.1074/jbc.M106344200 11546810

[72] Moya M Benet M Guzman C Tolosa L Garcia-Monzon C Pareja E. Foxa1 reduces lipid accumulation in human hepatocytes and is down-regulated in nonalcoholic fatty liver. PLoS One (2012) 7(1):e30014. doi: 10.1371/journal.pone.0030014 22238690 PMC3253125

[73] Weiss TS Lupke M Ibrahim S Buechler C Lorenz J Ruemmele P. Attenuated lipotoxicity and apoptosis is linked to exogenous and endogenous augmenter of liver regeneration by different pathways. PLoS One (2017) 12(9):e0184282. doi: 10.1371/journal.pone.0184282 28877220 PMC5587239

[74] Kurtz CL Peck BC Fannin EE Beysen C Miao J Landstreet SR. Microrna-29 fine-tunes the expression of key Foxa2-activated lipid metabolism genes and is dysregulated in animal models of insulin resistance and diabetes. Diabetes (2014) 63(9):3141–8. doi: 10.2337/db13-1015 PMC4141370 24722248

[75] Adler-Wailes DC Alberobello AT Ma X Hugendubler L Stern EA Mou Z. Analysis of variants and mutations in the human winged helix Foxa3 gene and associations with metabolic traits. Int J Obes (Lond) (2015) 39(6):888–92. doi: 10.1038/ijo.2015.17 PMC4462767 25672906

[76] Liu C Zhou B Meng M Zhao W Wang D Yuan Y. Foxa3 induction under endoplasmic reticulum stress contributes to non-alcoholic fatty liver disease. J Hepatol (2021) 75(1):150–62. doi: 10.1016/j.jhep.2021.01.042 33548387

[77] Gerin I Bommer GT Lidell ME Cederberg A Enerback S Macdougald OA. On the role of fox transcription factors in adipocyte differentiation and insulin-stimulated glucose uptake. J Biol Chem (2009) 284(16):10755–63. doi: 10.1074/jbc.M809115200 PMC2667763 19244248

[78] Fujimori K Amano F. Forkhead transcription factor Foxa1 is a novel target gene of C/Ebpbeta and suppresses the early phase of adipogenesis. Gene (2011) 473(2):150–6. doi: 10.1016/j.gene.2010.12.002 21167261

[79] Wolfrum C Shih DQ Kuwajima S Norris AW Kahn CR Stoffel M. Role of foxa-2 in adipocyte metabolism and differentiation. J Clin Invest (2003) 112(3):345–56. doi: 10.1172/jci18698 PMC166300 12865419

[80] Xu L Panel V Ma X Du C Hugendubler L Gavrilova O. The winged helix transcription factor Foxa3 regulates adipocyte differentiation and depot-selective fat tissue expansion. Mol Cell Biol (2013) 33(17):3392–9. doi: 10.1128/MCB.00244-13 PMC3753856 23798556

[81] Ma X Xu L Mueller E. Calorie hoarding and thrifting: Foxa3 finds a way. Adipocyte (2015) 4(4):325–8. doi: 10.1080/21623945.2015.1028700 PMC4573183 26451291

[82] Kanaki M Kardassis D. Regulation of the human lipoprotein lipase gene by the forkhead box transcription factor Foxa2/Hnf-3beta in hepatic cells. Biochim Biophys Acta Gene Regul Mech (2017) 1860(3):327–36. doi: 10.1016/j.bbagrm.2017.01.007 28126606

[83] Kanaki M Tiniakou I Thymiakou E Kardassis D. Physical and functional interactions between nuclear receptor lxralpha and the forkhead box transcription factor Foxa2 regulate the response of the human lipoprotein lipase gene to oxysterols in hepatic cells. Biochim Biophys Acta Gene Regul Mech (2017) 1860(8):848–60. doi: 10.1016/j.bbagrm.2017.05.007 28576574

[84] Wolfrum C BD Luca E Stoffel M. Insulin regulates the activity of forkhead transcription factor hnf-3beta/Foxa-2 by akt-mediated phosphorylation and Nuclear/Cytosolic localization. Proc Natl Acad Sci U.S.A. (2003) 100(20):11624–9. doi: 10.1073/pnas.1931483100 PMC208808 14500912

[85] von Meyenn F Porstmann T Gasser E Selevsek N Schmidt A Aebersold R. Glucagon-induced acetylation of Foxa2 regulates hepatic lipid metabolism. Cell Metab (2013) 17(3):436–47. doi: 10.1016/j.cmet.2013.01.014 23416070

[86] Wolfrum C Stoffel M. Coactivation of Foxa2 through pgc-1beta promotes liver fatty acid oxidation and Triglyceride/Vldl secretion. Cell Metab (2006) 3(2):99–110. doi: 10.1016/j.cmet.2006.01.001 16459311

[87] Bose SK Kim H Meyer K Wolins N Davidson NO Ray R. Forkhead box transcription factor regulation and lipid accumulation by hepatitis c virus. J Virol (2014) 88(8):4195–203. doi: 10.1128/JVI.03327-13 PMC3993747 24478438

[88] Tahri-Joutey M Andreoletti P Surapureddi S Nasser B Cherkaoui-Malki M Latruffe N. Mechanisms mediating the regulation of peroxisomal fatty acid beta-oxidation by pparalpha. Int J Mol Sci (2021) 22(16):8969. doi: 10.3390/ijms22168969 34445672 PMC8396561

[89] Ibrahim S Dayoub R Krautbauer S Liebisch G Wege AK Melter M. Bile acid-induced apoptosis and bile acid synthesis are reduced by over-expression of augmenter of liver regeneration (Alr) in a Stat3-dependent mechanism. Exp Cell Res (2019) 374(1):189–97. doi: 10.1016/j.yexcr.2018.11.023 30500391

[90] McDaniel K Meng F Wu N Sato K Venter J Bernuzzi F. Forkhead box A2 regulated biliary heterogeneity and senescence during cholestatic liver injury. Hepatology (2017) 65(2):544–59. doi: 10.1002/hep.28831 PMC5258713 27639079

[91] Bochkis IM Rubins NE White P Furth EE Friedman JR Kaestner KH. Hepatocyte-specific ablation of Foxa2 alters bile acid homeostasis and results in endoplasmic reticulum stress. Nat Med (2008) 14(8):828–36. doi: 10.1038/nm.1853 PMC4095974 18660816

[92] Bochkis IM Schug J Rubins NE Chopra AR O'Malley BW Kaestner KH. Foxa2-dependent hepatic gene regulatory networks depend on physiological state. Physiol Genomics (2009) 38(2):186–95. doi: 10.1152/physiolgenomics.90376.2008 PMC2712224 19417011

[93] Bochkis IM Shin S Kaestner KH. Bile acid-induced inflammatory signaling in mice lacking Foxa2 in the liver leads to activation of mtor and age-onset obesity. Mol Metab (2013) 2(4):447–56. doi: 10.1016/j.molmet.2013.08.005 PMC3855091 24327960

[94] Kain J Wei X Reddy NA Price AJ Woods C Bochkis IM. Pioneer factor Foxa2 enables ligand-dependent activation of type ii nuclear receptors fxr and lxralpha. Mol Metab (2021) 53:101291. doi: 10.1016/j.molmet.2021.101291 34246806 PMC8350412

[95] Lebeaupin C Vallee D Hazari Y Hetz C Chevet E Bailly-Maitre B. Endoplasmic reticulum stress signalling and the pathogenesis of non-alcoholic fatty liver disease. J Hepatol (2018) 69(4):927–47. doi: 10.1016/j.jhep.2018.06.008 29940269

[96] Dayoub R Groitl P Dobner T Bosserhoff AK Schlitt HJ Weiss TS. Foxa2 (Hnf-3beta) regulates expression of hepatotrophic factor alr in liver cells. Biochem Biophys Res Commun (2010) 395(4):465–70. doi: 10.1016/j.bbrc.2010.04.023 20382118

[97] Aghadi M Elgendy R Abdelalim EM. Loss of Foxa2 induces er stress and hepatic steatosis and alters developmental gene expression in human ipsc-derived hepatocytes. Cell Death Dis (2022) 13(8):713. doi: 10.1038/s41419-022-05158-0 35973994 PMC9381545

[98] Thymiakou E Kardassis D. Novel mechanism of transcriptional repression of the human atp binding cassette transporter A1 gene in hepatic cells by the winged Helix/Forkhead box transcription factor A2. Biochim Biophys Acta (2014) 1839(6):526–36. doi: 10.1016/j.bbagrm.2014.04.021 24807696

[99] Wolfrum C Howell JJ Ndungo E Stoffel M. Foxa2 activity increases plasma high density lipoprotein levels by regulating apolipoprotein m. J Biol Chem (2008) 283(24):16940–9. doi: 10.1074/jbc.M801930200 18381283

[100] Li Y Xu Y Jadhav K Zhu Y Yin L Zhang Y. Hepatic forkhead box protein A3 regulates apoa-I (Apolipoprotein a-I) expression, cholesterol efflux, and atherogenesis. Arterioscler Thromb Vasc Biol (2019) 39(8):1574–87. doi: 10.1161/ATVBAHA.119.312610 PMC6656627 31291759

[101] Wolf G. Brown adipose tissue: The molecular mechanism of its formation. Nutr Rev (2009) 67(3):167–71. doi: 10.1111/j.1753-4887.2009.00184.x 19239631

[102] Li J Jiang R Cong X Zhao Y. Ucp2 gene polymorphisms in obesity and diabetes, and the role of Ucp2 in cancer. FEBS Lett (2019) 593(18):2525–34. doi: 10.1002/1873-3468.13546 31330574

[103] Ma X Xu L Gavrilova O Mueller E. Role of forkhead box protein A3 in age-associated metabolic decline. Proc Natl Acad Sci U.S.A. (2014) 111(39):14289–94. doi: 10.1073/pnas.1407640111 PMC4191794 25225406

[104] Whitton H Singh LN Patrick MA Price AJ Osorio FG Lopez-Otin C. Changes at the nuclear lamina alter binding of pioneer factor Foxa2 in aged liver. Aging Cell (2018) 17(3):e12742. doi: 10.1111/acel.12742 29484800 PMC5946061

[105] Khalifa O Al-Akl NS Errafii K Arredouani A. Exendin-4 alleviates steatosis in an in vitro cell model by lowering Fabp1 and Foxa1 expression *Via* the wnt/-catenin signaling pathway. Sci Rep (2022) 12(1):2226. doi: 10.1038/s41598-022-06143-5 35140289 PMC8828858

[106] Chen Q Yang X Zhang H Kong X Yao L Cui X. Metformin impairs systemic bile acid homeostasis through regulating Sirt1 protein levels. Biochim Biophys Acta Mol Cell Res (2017) 1864(1):101–12. doi: 10.1016/j.bbamcr.2016.10.020 27816442

[107] Dai X Cheng H Chen X Li T Zhang J Jin G. Foxa1 is prognostic of triple negative breast cancers by transcriptionally suppressing Sod2 and Il6. Int J Biol Sci (2019) 15(5):1030–41. doi: 10.7150/ijbs.31009 PMC6535797 31182923

[108] Parolia A Cieslik M Chu SC Xiao L Ouchi T Zhang Y. Distinct structural classes of activating Foxa1 alterations in advanced prostate cancer. Nature (2019) 571(7765):413–8. doi: 10.1038/s41586-019-1347-4 PMC6661908 31243372

[109] Motallebipour M Ameur A Reddy Bysani MS Patra K Wallerman O Mangion J. Differential binding and Co-binding pattern of Foxa1 and Foxa3 and their relation to H3k4me3 in Hepg2 cells revealed by chip-seq. Genome Biol (2009) 10(11):R129. doi: 10.1186/gb-2009-10-11-r129 19919681 PMC3091322

